# Particle-size dependent bactericidal activity of magnesium oxide against *Xanthomonas perforans* and bacterial spot of tomato

**DOI:** 10.1038/s41598-019-54717-7

**Published:** 2019-12-06

**Authors:** Y. Y. Liao, A. Strayer-Scherer, J. C. White, R. De La Torre-Roche, L. Ritchie, J. Colee, G. E. Vallad, J. Freeman, J. B. Jones, M. L. Paret

**Affiliations:** 1 Plant Pathology Department, University of Florida, Gainesville, FL USA; 2 North Florida Research and Education Center, University of Florida, Quincy, FL USA; 3 Department of Entomology and Plant Pathology, Auburn University, Auburn, AL USA; 4 Department of Analytical Chemistry, The Connecticut Agricultural Experiment Station, New Haven, CT USA; 5 Gulf Coast Research and Education Center, University of Florida, Wimauma, FL USA; 6 Statistical Consulting Unit, Institute of Food and Agricultural Sciences, University of Florida, Gainesville, FL USA

**Keywords:** Antimicrobial resistance, Bacteriology

## Abstract

Bacterial spot, caused by *Xanthomonas* spp., is a highly destructive disease of tomatoes worldwide. Copper (Cu) bactericides are often ineffective due to the presence of Cu-tolerant strains. Magnesium oxide (MgO) is an effective alternative to Cu bactericides against *Xanthomonas* spp. However, the effects of particle size on bactericidal activity and fruit elemental levels are unknown. In this study, nano (20 nm) and micron (0.3 and 0.6 µm) size MgO particles were compared for efficacy. Nano MgO had significantly greater *in vitro* bactericidal activity against Cu-tolerant *X. perforans* than micron MgO at 25–50 µg/ml. In field experiments nano and micron MgO applied at 200 and 1,000 µg/ml were evaluated for disease control. Nano MgO at 200 µg/ml was the only treatment that consistently reduced disease severity compared to the untreated control. Inductively Coupled Plasma Optical Emission Spectroscopy revealed that nano MgO applications did not significantly alter Mg, Cu, Ca, K, Mn, P and S accumulation compared to fruits from the untreated plots. We demonstrated that although both nano MgO and micron MgO had bactericidal activity against Cu-tolerant strains *in vitro*, only nano MgO was effective in bacterial spot disease management under field conditions.

## Introduction

Tomato (*Solanum lycopersicum*) is an economically important crop in the United States and worldwide. Just in 2017, the total tomatoes production amounted to 12.5 million metric tons in the United States. The value of this crop totaled $1.67 billion dollars^1^. Bacterial spot is one of the most damaging diseases that can cause major yield reductions in the tomato market around the world, especially in where the high humidity and temperatures create a favorable environment^2–4^. Bacterial spot disease of tomato is caused by four distinct *Xanthomonas* species^5^. In Florida, which is the largest fresh market tomato producer in the United States^1^, *X. perforans* is the dominant causal agent of bacterial spot of tomato. Although the disease has been around since its discovery in South Africa in 1914^6^, effective disease management strategies for bacterial spot are currently limited. Given that Florida’s tomato production industry has a long history with bacterial spot disease, the pathogen has developed resistance toward bactericides including streptomycin^7,8^ and copper (Cu)^2,9^.

Cu-tolerant *Xanthomonas* strains were isolated in the 1960s, as grower’s noticed the diminishing efficacy of Cu bactericides^9^. Subsequently it was found that addition of ethylene- bis-dithiocarbamates (EBDC) to Cu bactericides provided better disease control and improved Cu solubility^9,10^. Since Cu-tolerant *Xanthomonas* strains are sensitive to Cu-EBDC, this option remains the standard treatment for tomato producers in Florida and elsewhere. However, when environmental conditions are optimal for disease development, even Cu-EBDC is ineffective against bacterial spot disease of tomato^11,12^.

Efforts to identify alternatives to Cu-EBDC have been extensive over the last two decades. For instance, bacteriophages have been extensively studied and are commercially available for managing bacterial spot disease^13–16^. However, bacteriophages are highly sensitive to environmental factors, which can decrease their efficacy^17,18^. Other available alternatives to Cu include plant defense activators such as acibenzolar S-methyl^15^, and plant-growth-promoting rhizobacteria that provide limited disease control under field conditions^19^. Currently, Florida’s tomato growers use a combination of Cu bactericides, plant defense activators, bio-fungicides, and cultural practices for bacterial spot management^2^. Importantly, nanoparticles including magnesium oxide (MgO), have been recently shown to have potential to be an alternative to Cu-EBDC^20–23^.

Nanoparticles by definition are materials smaller than or equal to 100 nanometers (nm)^24^ and are increasingly being developed for plant disease management^25,26^. Prior studies have demonstrated the improved antibacterial ability of metal and metal oxide nanoparticles compared to their micron particles^27–30^. Nair *et al*. demostrated that zinc oxide (ZnO) nanoparticles (40 nm) at 5 mM had bactericidal activity against both Gram-negative bacteria (*Escherichia coli*) and Gram-positive bacteria (*Staphylococcus aureus*), whereas the micron ZnO (1.2 μm) did not. In addition, Raghupathi *et al*. reported that ZnO nanoparticles (25 nm) had higher bactericidal activity against methicillin sensitive *Staphylococcus aureus* strains compared to the micron particles (0.2 µm). Furthermore, ZnO nanoparticles (~12 nm) at 4 mM had high antibacterial activity against methicillin resistant *S. aureus* strains and activity was similar to that against methicillin sensitive strains. The literature suggests that using smaller size particles of metal or metal oxides may lead to greater antibacterial activity toward antibiotic resistant bacterial pathogens affecting humans and animals^28,31,32^. For instance, Li *et al*. demonstrated that silver (Ag) nanoparticles (~120 nm) at 12.5 µg/ml were bactericidal to antibiotic-resistant *Neisseria gonorrhoeae*. Not only were nanoparticles known to have bactericidal activity against human pathogens, but several studies over past decade have demonstrated efficacy for plant disease management^20,22,33–35^. Bacterial spot disease of tomato is a model disease for many recent studies on the use of nanoparticles for management of Cu-tolerant bacterial strains^20–23^.

In a previous field study^23^, magnesium oxide nanoparticle (nano MgO) (US Research Nanomaterials, Inc., Houston, TX, USA) at 200 µg/ml significantly reduced disease severity of bacterial spot disease of tomato compared to the untreated control, whereas the grower’s standard (Cu-EBDC) did not significantly reduce disease severity compared to the untreated control in the field^23^ (*P* < 0.05). There was no negative impact on tomato fruit yield due to nano MgO treatments. Based on elemental analysis of fruit samples, nano MgO treatments did not impact accumulation of Mg, Cu, Ca, K, Mn, P, and S compared to the untreated control. Therefore, the study suggested that nano MgO could be a potential bactericide against bacterial spot disease of tomato without potential side effects due to elemental accumulation in the fruit.

Despite the fact that MgO is generally recognized as a safe (GRAS) compound^36^, multiple studies have described the fate of MgO nanoparticles in the environment as poorly understood^37–40^. In addition, regulatory guidelines for nanoparticles are still being developed by the U.S. Environmental Protection Agency (EPA)^41,42^ and Food and Drug Administration (FDA)^41^. Therefore, understanding the environmental fate and toxicology of nanoparticles such as MgO continues to be an important research area^43,44^.

Due to the potential concerns to the environmental fate of nanoparticles^45–47^, comparative studies into the efficacy of micron-size particles for disease control is a relevant area given that this approach may be more acceptable for commercialization under the current EPA regulations^41,48^. Unlike nanoparticles, micron-sized particles are considered lower risk to the environment. In addition, Sawai *et al*. demonstrated that micron-sized MgO (micron MgO) also has antibacterial activity against human pathogens. Since nano MgO showed antibacterial activity in the past^49,50^, the potential of micron MgO to provide a similar level of crop disease reduction in the field as the MgO nanoparticles should be explored.

We hypothesized that similar to nano MgO, micron MgO will have greater antibacterial properties compared to commercial micron size Cu *in vitro*, and will provide more effective control of bacterial spot of tomato in the field. The goal of this study was to determine if MgO particle size is critical to increased efficacy against bacterial spot disease of tomato. The objectives of this study were to (i) evaluate the efficacy of nano- and micron-size MgO against Cu-tolerant *X. perforans in vitro* and in the field, and (ii) to evaluate whether the different size of MgO material leads to different accumulation of Mg or other elements in the fruit.

## Results

### Bactericidal activity of different particle size of MgO compared with Cu bactericide

**Figure 1:**
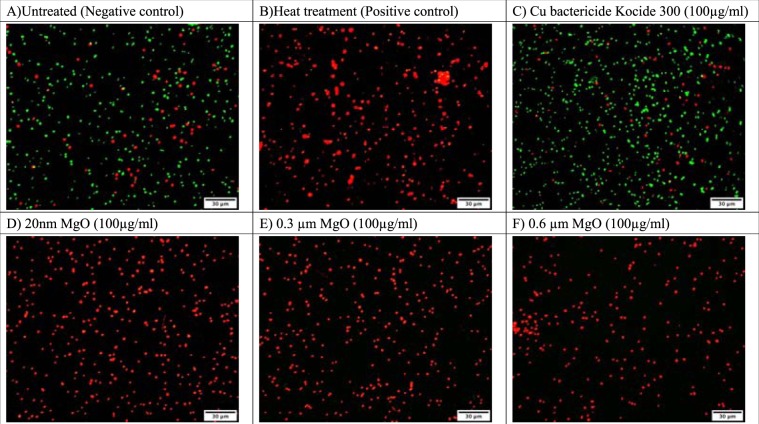
Viability assay of Cu-tolerant *X. perforans* GEV485 treated with 100 µg/ml MgO (20 nm, 0.3 µm, and 0.6 µm). Untreated, heated treated, and the Cu bactericide Kocide ® 3000 were used as controls. Cells stained with the LIVE/DEAD^®^ BacLight™ Bacterial Viability Kit green florescence indicate live cells and red florescence indicate dead cells. Micrographs were taken on a Nikon Eclipse Ti inverted microscope (Nikon, Melville, NY) at ×40 fluorescent optics using NIS-Elements imaging software (Ver. 3.0; Nikon).

The bactericidal activity was confirmed by the viability assay (Fig. 1A–F). Bacterial mortality was nearly 100% (red fluorescence) after treatment with 100 µg/ml MgO (20 nm, 0.3 and 0.6 µm) (Fig. 1D–F) for 4 h similar to the heat treated positive control (Fig. 1B). In comparison, the Cu bactericide (Kocide 3000) (Fig. 1C) had 80.7% alive cells (green fluorescence), which was similar to the untreated control (80% alive cells) (Fig. 1A).

### Effect of MgO particle size on *in vitro* growth of *X. perforans*

**Figure 2:**
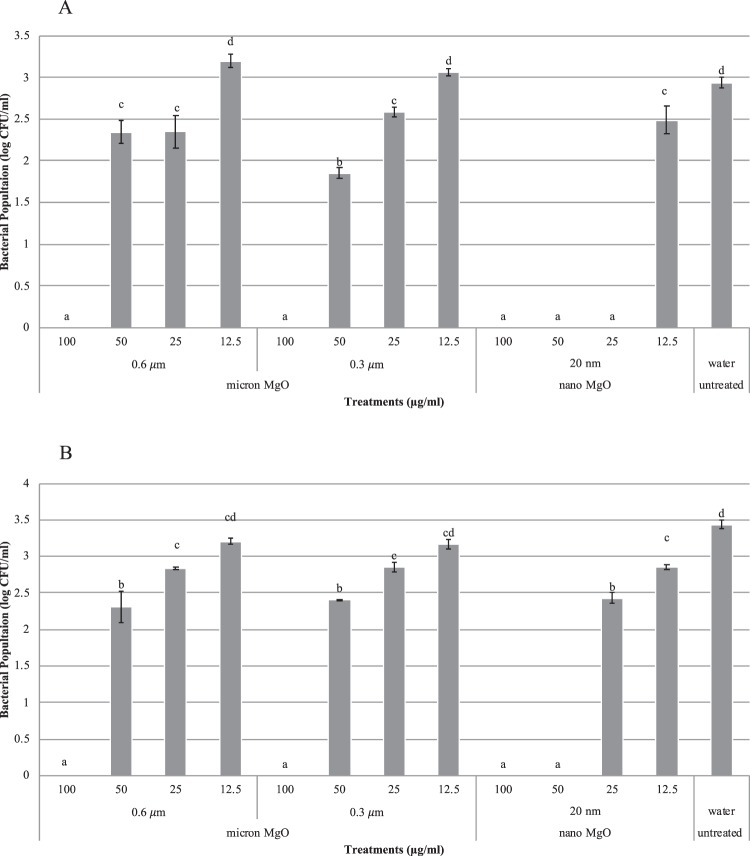
(**A**) *In vitro* inhibition of Cu-tolerant *Xanthomonas perforans* strain GEV485 following exposure to MgO particles for 4 h. Treatments were as follows: 20 nm, 0.3 μm and 0.6 μm MgO at 100, 50, 25, and 12.5 μg/ml. The treatments were compared with water control (UT) at incubation times of 4 h. Error bars indicate standard error of the mean. *P* value of 0.05 was used in the IBM SPSS using Student-Newman-Keuls statistical analysis. (**B**) Repeated *in vitro* inhibition of Cu-tolerant *Xanthomonas perforans* strain GEV485 following exposure to MgO particles for 4 h. Treatments were as follows: 20 nm, 0.3 μm and 0.6 μm MgO at 100, 50, 25, and 12.5 μg/ml.

Both nano and micron (20 nm, 0.3, and 0.6 µm) MgO had significant antimicrobial activity at 100 µg/ml against the Cu-tolerant strain, *X. perforans* GEV485 after 4 h (Fig. 2A,B). The minimum bactericidal concentration (MBC) for nano MgO against Cu-tolerant *X. perforans* strain was 25–50 µg/ml, whereas the MBC for micron MgO (0.3 and 0.6 µm) was 100 µg/ml (Fig. 2A,B).

### Comparison of field efficacy of MgO with Cu and Cu-EBDC for management of tomato bacterial spot

In the first field experiment, conducted during spring 2016 in Quincy, FL, plants that received either concentration of nano MgO (20 nm) (1,000 µg/ml or 200 µg/ml) had significantly less disease compared to the untreated control, but were not different from the other treatments (Table 1). Of the two larger size MgO particle sizes tested, only 0.3 µm MgO at 1,000 µg/ml significantly reduced disease severity compared to the untreated control. Both Cu bactericide and Cu-EBDC were not significantly different from the control. Treatment applications did not cause any phytotoxicity on the tomato plants (data not shown).

**Table 1:** Comparison of nano magnesium oxide (MgO) (20 nm MgO), micron MgO (0.3 and 0.6 µm MgO), copper (Kocide 3000), and with the grower standard (Cu-EBDC) for control of bacterial spot disease severity (area under disease progress curve - AUDPC) on tomato variety ‘BHN602’ in three field experiments in Quincy, FL.

Treatment	Rate (µg/ml)	AUDPC^y^ in different seasons^w^			
		2016 Spring		2016 Fall	
20 nm MgO	1,000	862.1	a^z^	770.6	a
20 nm MgO	200	857.3	a	767.2	a
0.3 µm MgO	1,000	859.5	a	898.1	a
0.3 µm MgO	200	919.6	ab	942.6	ab
0.6 µm MgO	1,000	946.7	ab	1004.1	ab
0.6 µm MgO	200	1023.4	ab	948.1	ab
Kocide 3000	2,100	1085.2	ab	1351.7	c
Cu-EBDC^x^		1085.4	ab	1144.3	bc
Water (Untreated)		1159.4	b	1155.7	bc

^z^Number with different character in the same column has significant difference (*P* value of = 0.05) based on Least Significant Difference statistical analysis using the IBM^®^ SPSS^®^ program.

^y^The area under disease progress curve (AUDPC) was calculated using the midpoint values of Horsfall-Barratt disease severity scale^70,71^.

^x^Cu-EBDC is composed of Kocide 3000 (2,100 µg/ml) and Penncozeb^®^ 75DF (1,200 µg/ml).

^w^The field trials were conducted in two seasons in Quincy, FL.

In the second trial during fall 2016 at Quincy, FL, (Table 1), both concentrations (1,000 and 200 µg/ml) of the nano-MgO (20 nm) significantly reduced disease severity compared to the untreated control, whereas the grower’s standard Cu-EBDC did not significantly reduce disease in the field trials compared to the untreated control (Table 1). Neither of the micron MgO (0.3 µm and 0.6 µm) treatments showed significant disease reduction compared to the untreated control in this trial (Table 1). No phytotoxicity was observed for any of the treatments in this experiment (data not shown). There were no significant impacts on total yield due to MgO treatments in both field trials (Table 2).

**Table 2:** Total and marketable yield in two field experiment following treatment of tomato plants ‘BHN602’ with nano magnesium oxide (MgO) (20 nm MgO), micron MgO (0.3 and 0.6 µm MgO), copper (Kocide 3000), and the grower standard (Cu-EBDC) in Quincy, FL.

Treatments	(µg/ml)	2016 Spring		2016 Fall	
		Total yield (kg/ha)	Marketable yield (%)	Total yield (kg/ha)	Marketable yield (%)
20 nm MgO	1,000	19,142 ± 159.5	93.10	47,536 ± 4,451.5	76.19
20 nm MgO	200	13,006 ± 413.7	86.32	47,745 ± 7,896.4	72.58
0.3 µm MgO	1,000	27,627 ± 971.3	90.05	53,429 ± 6,015.0	81.66
0.3 µm MgO	200	16,828 ± 599.8	87.17	63,023 ± 11,778.5	78.76
0.6 µm MgO	1,000	18,998 ± 246.6	89.57	59,241 ± 8,746.7	75.17
0.6 µm MgO	200	21,976 ± 766.7	87.93	53,726 ± 3,559.1	77.17
Kocide 3000	2,100	22,389 ± 943.9	89.13	58,744 ± 7,512.3	78.66
Cu-EBDC^z^		19,178 ± 759.0	91.81	42,719 ± 6,566.5	74.46
**Water** (untreated)		22,066 ± 587.6	86.43	55,908 ± 4,242.9	73.79
**Significance** (P = 0.05)^y^		NS	NS	NS	NS

^z^Cu-EBDC is composed of Kocide 3000 (2,100 μg/ml) and Penncozeb 75DF (1,200 μg/ml).

^y^None of the treatments had significant yield impact compared with the water treatment (P value of 0.05) based on Least Significant Difference statistical analysis using the IBM SPSS program; NS = not significant compared to Water (untreated).

### Accumulation of metals in harvested tomato fruits treated with MgO

In the spring 2016 trial (Table 3), there were no significant differences for any of the elements (Al, B, Ca, Cu, Fe, K, Mg, Mn, Mo, Na, P, S, and Zn) (Table S1) when comparing nano MgO (20 nm) treated fruit with the untreated controls. Similarly, fruit collected from micron MgO (0.3 µm and 0.6 µm) treated plots, at both concentrations of 0.3 µm MgO and 1,000 µg/ml of 0.6 µm MgO had no significant differences in elemental concentration compared to the untreated control. As for Cu bactericide (Kocide 3000) and the grower standard (Cu-EBDC), both treatments showed significantly higher Cu concentrations relative to the untreated control. The Cu bactericide treated fruits contain Cu that is twice as high (4.5 mg/kg higher) as the untreated fruit. For the fruit collected in fall 2016 trial (Tables 3), 1,000 µg/ml of 0.3 µm MgO treatment showed significantly higher Al content in the peel, with approximately 4 mg/kg more Al in dry weight compared to the untreated control (Table 3). However, Al did not significantly accumulate in either whole fruit or flesh for the 1,000 µg/ml of 0.3 µm MgO. Fruit receiving 1,000 µg/ml 0.3 µm MgO also contained significantly higher levels of Ca in the whole fruit (+8 mg/kg more in fresh weight) and peel (+0.043 mg/kg more in fresh weight) compared to the untreated control. The Cu-EBDC treatment showed significantly higher Ca content (+12.26 mg/kg more in fresh weight); values were at nearly two-fold of that accumulated in the untreated fruit (Table 3). Unlike the spring trial, Cu content of the fruit was not significantly impacted by the Kocide 3000 or Cu-EBDC treatment.

**Table 3:** Elemental accumulation in fruits (mg/kg) collected from tomato ‘BHN602’ in the fields treated with 200 and 1,000 µg/ml for 20 nm, 0.3 µm MgO and 0.6 µm MgO in comparison to Cu (Kocide 3000), the grower standard (Cu-EBDC) and the untreated (water) in Quincy, FL, USA from the harvest (7 days after the last application) in spring and fall 2016 trials. Besides whole fruit samples, flesh and peel samples were also collected separately.

		2016 Spring					2016 Fall				
		Whole					Flesh	Peel			Whole
		Al		Cu		Zn	Ca	Al		Ca	Ca
Treatments	(µg/ml)	dry^w^	fresh	dry	fresh	fresh	fresh	dry	fresh	fresh	fresh
20 nm MgO	1,000	7.21 a^z^	0.27 bc	3.75 a	0.15 a	0.72 a	11.65 b	0.41 a	0.004 a	24.75 ab	17.6 ab
20 nm MgO	200	4.81 a	0.19 ab	3.61 a	0.14 a	0.74 a	7.69 ab	0.40 a	0.003 a	21.54 ab	18.0 ab
0.3 µm MgO	1,000	5.69 a	0.21 ab	4.60 a	0.19 a	1.02 b	12.16 b	4.39 b	0.054 b	31.74 b	22.0 b
0.3 µm MgO	200	3.87 a	0.18 ab	3.75 a	0.17 a	0.90 ab	9.29 ab	ND^y^ a	ND a	28.19 ab	17.7 ab
0.6 µm MgO	1,000	2.58 a	0.11 ab	5.04 a	0.22 ab	0.97 ab	9.57 ab	0.59 a	0.006 a	25.04 ab	18.5 ab
0.6 µm MgO	200	10.7 b	0.40 c	4.28 a	0.16 a	0.76 a	8.45 ab	0.70 a	0.008 a	24.05 ab	17.7 ab
Kocide 3000		4.64 a	0.19 ab	9.12 c	0.38 c	0.88 ab	6.47 a	0.84 a	0.008 a	20.82 ab	14.2 a
Cu-EBDC^x^		4.30 a	0.17 ab	6.60 b	0.27 b	0.75 a	9.12 ab	0.89 a	0.007 a	29.56 b	18.6 ab
Water		6.78 a	0.30 bc	4.67 a	0.20 ab	0.82 ab	9.54 ab	0.20 a	0.001 a	17.30 a	14.2 a
Significance^v^			NS			NS	NS				

All the samples were evaluated with Inductively Coupled Plasma Optical Emission Spectroscopy (ICP-OES) for elemental accumulation.

^z^Within a column, different letters indicate significant difference compared to water treatment (*P* value of 0.05) based on Student-Newman-Keuls statistical analysis using the IBM® SPSS® program.

^y^ND is not-detected, concentration is below the limit of detection.

^x^Cu-EBDC is composed of Kocide 3000 (2,100 µg/ml) and Penncozeb^®^ 75DF (1,200 µg/ml).

^w^Dry/fresh is the concentration of metal in dry weight/ fresh weight tomato

^v^NS = not significant compared to water (untreated).

## Discussion

In this study, we compared nano and micron size MgO for *in vitro* bactericidal activity to copper-tolerant *X. perforans* bacterial cells. We demonstrated in the viability assay that both nano (20 nm) and micron (0.3 µm and 0.6 µm) MgO at concentrations as low as 100 µg/ml had high bactericidal activity (100% percent reduction) after 4 h. In comparison, Cu bactericide (Kocide 3000) was similar (19.3% mortality) to the untreated control (20% mortality). These results indicate that both nano (20 nm) and micron (0.3 µm and 0.6 µm) MgO at 100 µg/ml were more effective against Cu-tolerant *X. perforans* compared to Cu bactericide. However, in the *in vitro* assay, the MBC of nano-MgO (20 nm) against Cu-tolerant *X. perforans* was 25–50 µg/ml, whereas the MBC of micron (0.3 µm and 0.6 µm) MgO was 100 µg/ml. Therefore, this experiment demonstrated that nano MgO (20 nm) had significantly greater bactericidal activity compared to micron (0.3 µm and 0.6 µm) MgO (*P < *0.05) *in vitro*. This finding was consistent with the results of Huang *et al*. that the MgO antibacterial activity is size dependent *in vitro*. Huang *et al*. demonstrated that the bactericidal efficacy against *Bacillus subtilis* var. *niger* increased from 93% to 97%, when the MgO nanoparticle size decreased from 69 nm to 26 nm. However, that report focused on the MgO nanoparticles less than 100 nm, whereas in this study, we focused on evaluating antibacterial activity of nano (20 nm) and micron MgO (0.3 µm and 0.6 µm) against Cu-tolerant *X. perforans*.

Since both nano and micron MgO showed bactericidal activity against Cu-tolerant *X. perforans in vitro*, we compared the effectiveness of these materials with Cu bactericide and grower standard Cu-EBDC in the field. According to the field experiments, only nano MgO (20 nm) as low at 200 µg/ml provided significant disease reduction consistently compared with the untreated control (*P < *0.05) in both 2016 Spring and Fall field trials in Quincy, FL. In 2016 Fall field trial, nano MgO (20 nm) even provided greater disease control than the grower’s standard Cu-EBDC (*P < *0.05). Although both nano (20 nm) and micron (0.3 µm and 0.6 µm) MgO had antibacterial activity *in vitro*, the field trial experiments showed that only nano MgO (20 nm) could significantly reduce disease severity in the field.

In the past decade, nano MgO particles have been shown to have antimicrobial activity against several mammalian pathogens^51,52^. Additionally, MgO nanoparticles (~50 ± 10 nm) at concentrations as low as 100 μg/ml resulted in high inhibition rates of fungal spore germination of several fungal plant pathogens including *Alternaria alternata*, *Fusarium oxysporum*, *Rhizopus stolonifer*, and *Mucor plumbeus*^53^. Recently, a limited number of studies have explored utilizing Mg nanomaterials to manage bacterial pathogens^23,54^. Based on these studies, antibacterial mechanisms were proposed for MgO nanoparticle against bacterial cell at the nano-bio interface. Liao *et al*. used transmission electron microscopy (TEM) to show that MgO nanoparticles could cause membrane damage on *X. perforans*. Cai *et al*. suggested that reactive oxygen species (ROS) accumulation could play an important role for the antibacterial efficacy of MgO, inducing DNA damage, against *Ralstonia solanacearum*. These findings demonstrate the potential of utilizing MgO nanoparticles to manage plant pathogens in agriculture systems. Furthermore, MgO is a more sustainable treatment option since, unlike Cu, it is not on the list of the EPA’s Toxic Release Inventory (TRI) Program or in the Integrated Risk Information System^36^. Last but not least, by using MgO nanoparticles as alternatives to Cu bactericide would reduce the selective pressure on the developing Cu-tolerant *X. perforans* in the field.

The fate of engineered nanomaterials is a concern along with approaches involving material release into the environment. Due to the use of MgO in medical field such as cancer research, studies investigating the toxicity of MgO nanoparticles toward mammals have been conducted^55,56^. Lai *et al*. demonstrated that when compared to ZnO and TiO_2_, MgO nanoparticles ( < 50 nm) had the least toxicity to human neural cells. Gerloff *et al*. evaluated the cytotoxicity and oxidative DNA damage effect of MgO nanoparticles (8 nm) in human intestinal Caco-2 cells. The study showed that MgO nanoparticles (8 nm) did not cause significant membrane damage on Caco-2 cells in the cytotoxicity study. However, in order to fit the National Nanotechnology Initiative (NNI), which supports responsible development of nanotechnology, studies on the fate and effects of nanoparticle MgO in the environment are limited^37–40^; such work should be done to ensure the sustainability of such approaches as part of nano-enabled agriculture.

The metallic elemental composition derived from nano and micron MgO treatments in fruit is of relevant concern to the research community and general public. As shown in this study, there were no significant differences for any of the elements (Al, B, Ca, Cu, Fe, K, Mg, Mn, Mo, Na, P, S, and Zn) (Table S1) when comparing nano MgO (20 nm) treated fruit with the untreated controls. This finding is consistent with our previous study in which the nano MgO treatments did not impact accumulation of elemental concentration in the fruit compared to the untreated control^23^. In 2016 Spring trial (Table 3), similar to nano MgO (20 nm), micron MgO (0.3 µm and 0.6 µm) did not alter the elemental composition compared to fruits in the untreated control. However, fruits collected from Cu bactericide (Kocide 3000) and the grower standard (Cu-EBDC) treated plots showed significantly higher Cu concentrations (+0.2 mg/kg in fresh weight) relative to the untreated control. The study by Liao *et al*. also revealed that Cu-EBDC treated fruits had significantly greater Cu accumulation based on fresh weight more than the untreated control. Though consumption of tomato treated with Cu bactericide might lead to more Cu exposure, the concentration is still within the daily dietary limit^57,58^. In addition, unlike the spring trial, the Cu concentration of fruit was not significantly impacted by the Kocide 3000 or Cu-EBDC treatment in the fall trial. Former studies suggest that different environmental conditions such as crop variety, heavy metal exposure time, and location may affect Cu accumulation or elemental composition in crops^59–61^. Although applying Cu bactericides may increase Cu content in the fruit slightly without immediate risk to the consumer, field runoff containing Cu still pose ecological risk to non-target aquatic organisms^62–64^. In addition, Cu accumulation in the soil will potential cause phytotoxicity to tomato plants^65,66^. Thus it is still critical to find effective alternatives against bacterial spot to avoid intense Cu application in agriculture system.

In conclusion, although both micron (0.3 µm and 0.6 µm) and nano (20 nm) MgO have bactericidal activity against Cu-tolerant *X. perforans in vitro*, whereas micron MgO did not significantly reduce disease severity as effectively as nano MgO in the field. Importantly, the efficacy of MgO against bacterial spot disease of tomato is size dependent. Nano MgO bactericide still has great potential to become an alternative to Cu bactericides against bacterial spot disease of tomato as long as regulatory clearance can be obtained.

## Materials and Methods

### Bacterial strains and storage

*X. perforans* strain GEV485 (Cu-tolerant), isolated from tomato in Florida, was used in this study. Bacterial cells from pure cultures of these strains were suspended in sterile 30% glycerol solution and stored at −80 °C. Prior to use, bacteria were grown on nutrient agar (NA) medium (BBL, Becton Dickinson and Co., Cockeysville, MD) at 28 °C and were transferred every 24 to 48 h. Bacterial cells were collected from cultures grown on NA for 24 h, suspended in 0.01 M MgSO_4_, and the suspensions were adjusted to A_600_ = 0.3 at λ = 600 nm (~5 × 10^8^ CFU/ml).

### Magnesium oxide particles

Magnesium oxide particles (MgO, 99 + %, 20 nm, 0.3 and 0.6 µm) were purchased in powder form from US Research Nanomaterials, Inc. (Houston, TX, USA). The powder was suspended in autoclaved deionized water, and sonicated with a Branson B-22-4 Ultrasonic Cleaner (Danbury, CT, USA) for 10 min in sterile deionized water. The suspension was adjusted to 1,000 µg/ml and used as a stock suspension.

### *In vitro* experiment evaluating minimum inhibitory concentration

*X. perforans* strain GEV485 (Cu-tolerant) was cultured from −80 °C storage and was suspended in sterile tap water and suspensions were diluted to 10^5^ CFU/ml. Twenty micoliters of the bacterial suspension were transferred to 2 mL of MgO (20 nm, 0.3 and 0.6 µm) at different concentrations (100, 50, 25, and 12.5 µg/ml) in glass tubes. Sterile tap water served as the control. The tubes were incubated on a shaker (200 rpm) at 28 °C. Fifty microliters were sampled from each tube and plated on nutrient agar. Bacterial colonies were counted on each plate and converted to colony forming units (CFU)/ml.

### Viability assay evaluating bactericidal activity

*X. perforans* strain GEV485 was used for the viability assay. Bacterial cells were incubated in nutrient broth (BBL, Becton Dickinson and Co., Cockeysville, MD) at 28 °C on a shaker at 300 rpm for 16 h to log phase. Bacterial cells were pelleted by centrifugation (16,872 × *g* for 10 min) and resuspended in 0.01 M MgSO_4_, and the suspensions were adjusted to A_600_ = 0.3 at λ = 600 nm (~5 × 10^8^ CFU/ml). Then 4.5 mL of the bacterial suspension were transferred to 500 μl of the following treatments in sterile glass tubes: 3 particle sizes of MgO (i.e., 20 nm, 0.3, or 0.6 µm), Cu bactericide (Kocide® 3000 (DuPont, Wilmington, DE)) at 1,000 µg/ml. Sterilized tap water served as the control. The tubes were incubated on a shaker (300 rpm) at 28 °C for 4 h. After washing with 1 mL 0.85% NaCl twice, 1 ml samples from each tube were stained using the LIVE/DEAD BacLight Bacterial Viability kit (L7007, Molecular Probes, Invitrogen). The stain was a mixture of 1.5 ml Component A (SYTO 9 dye, 1.67 mM/Propidium iodide, 1.67 mM) with 1.5 ml Component B (SYTO 9 dye, 1.67 mM/Propidium iodide, 18.3 mM). Following addition of the stain, the sample was incubated in darkness for 15 min at room temperature. Micrographs were taken on a Nikon Eclipse Ti inverted microscope (Nikon, Melville, NY) at ×40 fluorescent optics using NIS-Elements imaging software (Ver. 3.0; Nikon, Melville, NY). The dead cell/ all cell ratio was calculated by ImageJ^67^.

### Field experiments

Different MgO particle sizes were tested against bacterial spot disease of tomato in two field trials (Season one: 18 Apr 2016 to 13 June 2016 in Quincy, FL; Season two: 13 Sep 2016 to 18 Oct 2016 in Quincy, FL). Each treatment had four replications consisting of 15 BHN 602 variety tomato plants. The plots were arranged in a completely randomized block design. Experimental plots were spaced 1.8 m apart and plants were spaced 50.8 cm within the row^68^. Fertilizers were applied to plots based on soil type and cooperative extension recommendations^69^. Tomato transplants were grown in 128-cell containers under greenhouse conditions before transplanting. After transplanting, the treatments were sprayed on the foliar parts of tomatoes at the rate of 1.2 liter for four plots one week prior to bacterial inoculation. The treatments consisted of 200 and 1,000 µg/ml of 20 nm, 0.3 µm, and 0.6 µm of MgO suspension, sonicated in Branson B-22-4 Ultrasonic Cleaner (Danbury, CT, USA) for 10 min, with constant shaking while applying; Kocide 3000 (2.1 g/liter), the grower standard Kocide 3000 (2.1 g/liter) in combination with Penncozeb 75DF (1.2 g/liter; United Phosphorus, Inc., King of Prussia, PA) (Cu-EBDC) and an untreated control (water). To ensure adequate disease development in the field plots, a suspension of Cu-tolerant *X. perforans* strain GEV485 was adjusted to 5 × 10^8^ CFU/ml in deionized water and was applied to the foliage in the field by spraying the 1^st^, 8^th^, and 15^th^ plant in each plot. One liter of each treatment was applied to each plot weekly with CO_2_ pressurized spray boom with five nozzles until one week before fruit harvest. Plants were assessed for bacterial spot disease severity and phytotoxicity using the Horsfall-Barratt disease severity scale^70^ every week after inoculation until harvest. The area under disease progress curve (AUDPC) was then calculated^71^. There were four replications per treatment and the experiment was conducted three times. Twelve out of fifteen plants, excluding the two towards the two ends of plots, were harvested for assessing the yield. Mature green or early breaker stage fruit were harvested and graded by USDA standards^72^. At least two harvests were made for each field experiment, which is common for fresh market tomato production in Florida.

### Elemental analysis of the fruits

At harvest, five medium-sized mature-green stage fruits, with diameters between 5.72 and 6.43 cm according to the USDA standards^72–74^ were collected from each of the treatments. The fruit were harvested from outside of the canopy from the first and the last plants of each of four plots from the 2016 Spring and Fall Quincy trial at 7 days after final application. The harvested fruit were hand-washed and sent to Department of Analytical Chemistry, The Connecticut Agricultural Experiment Station, New Haven, CT, USA. Four to eight grams of fresh tomato fruit with peel and flesh, and four to eight grams each of peel only and flesh only samples were dried in an electric oven at 70 °C for 48 h. Dried samples were pre-digested overnight with 2 ml of concentrated nitric acid and 2 ml of H_2_O_2_. After the pre-digestion step, these samples were digested at 115 °C for 45 min and then cooled to room temperature. The samples were filtered through cotton plugs and the volume was adjusted to 50 ml. The samples were stored at room temperature until analysis. Al, B, Ca, Cu, Fe, K, Mg, Mn, Mo, Na, P, S, and Zn concentrations were determined by Inductively Coupled Plasma Optical Emission Spectroscopy (ICP-OES) using the Atom Scan 16 (Thermo-Jarrell Ash, Franklin, MA, USA). Analysis was performed following the methods described in previous studies^75–77^.

### Statistical analysis

The data collected from the *in vitro* assays and field experiments were evaluated for statistical significance using ANOVA followed by pair-wise comparisons using either the Least Significant Difference (LSD) for field studies, and the Student Newman-Keuls (SNK) method for *in vitro* and elemental accumulation experiments in IBM^®^ SPSS^®^ Statistics Version 22. A p-value of 0.05 was used to evaluate significance.

## Supplementary information

Dataset 1
