# Supplementary material for: Particle-size dependent bactericidal activity of magnesium oxide against Xanthomonas perforans and bacterial spot of tomato
Source: Sci Rep. 2019 Dec 6;9:18530. doi: 10.1038/s41598-019-54717-7 (PMC6898373; doi:10.1038/s41598-019-54717-7)
Supplement: Supplementary file 1 — Dataset 1 [file 41598_2019_54717_MOESM1_ESM.docx]

**Supplementary File**

**Particle-size dependent bactericidal activity of magnesium oxide against *Xanthomonas perforans* and bacterial spot of tomato**

Liao, YY.^1,2^, Strayer-Scherer, A.^1,2,3^, White, J.C.^4^, De La Torre-Roche, R. ^4^, Ritchie, L.^2^, Colee, J.^6^, Vallad G. E^5^., Freeman, J.^2^, Jones, J. B.^1^ and Paret, M. L.^1,2^

^1^Department of Plant Pathology, University of Florida, Gainesville, FL, USA

^2^North Florida Research and Education Center, University of Florida, Quincy, FL, USA

^3^Department of Entomology and Plant Pathology, Auburn University, Auburn, AL, USA

^4^Department of Analytical Chemistry, The Connecticut Agricultural Experiment Station, New Haven, CT, USA

^5^Gulf Coast Research and Education Center, University of Florida, Wimauma, FL, USA

^6^ Statistical Consulting Unit, Institute of Food and Agricultural Sciences, University of Florida, Gainesville, FL, USA

Supplementary Table S1. Elemental accumulation in fruits (mg/kg) collected from tomato ‘BHN602’ in the fields treated with 200 and 1,000 µg/ml for 20nm, 0.3 µm MgO and 0.6 µm MgO in comparison to Cu (Kocide 3000), the grower standard (Cu-EBDC) and the untreated (water) in Quincy, FL, USA from the harvest (7 days after the last application) in spring and fall 2016 trials. Besides whole fruit samples, flesh and peel samples were also collected separately. All the samples were evaluated with Inductively Coupled Plasma Optical Emission Spectroscopy (ICP-OES) for elemental accumulation.

|  | Rate | | Elemental accumulation in fruit (mg/kg FW)^x^ | | | | | | | | | | | | | | | | | | | | | | | | |
| --- | --- | --- | --- | --- | --- | --- | --- | --- | --- | --- | --- | --- | --- | --- | --- | --- | --- | --- | --- | --- | --- | --- | --- | --- | --- | --- | --- |
| Treatments | (µg/ml) | | Al | | B | | Ca | | Cu | Fe | | K | | Mg | | Mn | | Mo | | Na | | P | | S | | Zn | |
| 2016 Spring Trial  Whole fruit | | | | | | | | | | | | | | | | | | | | | | | | | | | |
| 20nm MgO | 1,000 | | 0.274 | | 0.465 | | 56.402 | | 0.148 | 1.236 | | 1443.802 | | 64.035 | | 0.689 | | 0.032 | | 13.966 | | 93.339 | | 58.566 | | 0.720 | |
| 20nm MgO | 200 | | 0.189 | | 0.466 | | 45.400 | | 0.144 | 1.333 | | 1457.750 | | 62.690 | | 0.666 | | 0.028 | | 12.409 | | 89.774 | | 61.689 | | 0.737 | |
| 0.3µm MgO | 1,000 | | 0.207 | | 0.486 | | 45.013 | | 0.186 | 1.486 | | 1529.349 | | 70.379 | | 0.680 | | 0.025 | | 13.220 | | 111.176 | | 65.479 | | 1.023 | |
| 0.3µm MgO | 200 | | 0.180 | | 0.505 | | 40.123 | | 0.174 | 1.647 | | 1649.591 | | 72.523 | | 0.733 | | 0.029 | | 15.392 | | 110.473 | | 70.508 | | 0.900 | |
| 0.6µm MgO | 1,000 | | 0.112 | | 0.518 | | 39.237 | | 0.220 | 1.536 | | 1652.794 | | 75.580 | | 0.692 | | 0.035 | | 14.293 | | 120.187 | | 67.146 | | 0.973 | |
| 0.6µm MgO | 200 | | 0.397 | | 0.468 | | 51.258 | | 0.162 | 1.272 | | 1348.059 | | 64.754 | | 0.631 | | 0.030 | | 14.151 | | 93.777 | | 56.012 | | 0.761 | |
| Kocide 3000 |  | | 0.193 | | 0.551 | | 56.048 | | 0.348* | 1.309 | | 1537.101 | | 74.807 | | 0.764 | | 0.026 | | 15.005 | | 107.432 | | 64.611 | | 0.805 | |
| Cu-EBDC |  | | 0.169 | | 0.502 | | 45.317 | | 0.266 | 1.252 | | 1469.901 | | 69.038 | | 0.734 | | 0.030 | | 16.532 | | 99.747 | | 59.314 | | 0.755 | |
| Water |  | | 0.300 | | 0.542 | | 45.576 | | 0.205 | 1.455 | | 1632.456 | | 77.498 | | 0.730 | | 0.027 | | 16.473 | | 110.226 | | 66.061 | | 0.817 | |
| Significance^y^ |  | | NS | | NS | | NS | | Yes | NS | | NS | | NS | | NS | | NS | | NS | | NS | | NS | | NS | |
| 2016 Fall Trial  Whole fruit | | | | | | | | | | | | | | | | | | | | | | | | | | | |
| 20nm MgO | 1,000 | ND^w^ | | 0.131 | | 17.551 | | 0.089 | | 0.788 | 525.476 | | 22.001 | | 0.155 | | 0.005 | | 2.277 | | 34.143 | | 22.238 | | 0.232 | |  |
| 20nm MgO | 200 | ND | | 0.185 | | 18.003 | | 0.113 | | 0.843 | 677.363 | | 30.775 | | 0.198 | | 0.007 | | 2.904 | | 42.409 | | 28.413 | | 0.304 | |  |
| 0.3µm MgO | 1,000 | 0.003 | | 0.188 | | 22.024* | | 0.137 | | 0.881 | 634.152 | | 31.746 | | 0.245 | | 0.011 | | 3.195 | | 47.896 | | 29.430 | | 0.353 | |  |
| 0.3µm MgO | 200 | 0.690 | | 0.149 | | 17.747 | | 0.099 | | 0.635 | 506.041 | | 23.984 | | 0.168 | | 0.010 | | 2.521 | | 34.640 | | 22.646 | | 0.261 | |  |
| 0.6µm MgO | 1,000 | ND | | 0.172 | | 18.491 | | 0.104 | | 0.597 | 633.862 | | 27.606 | | 0.173 | | 0.006 | | 2.842 | | 36.734 | | 24.995 | | 0.271 | |  |
| 0.6µm MgO | 200 | 0.006 | | 0.157 | | 17.716 | | 0.098 | | 0.794 | 612.069 | | 26.769 | | 0.174 | | 0.007 | | 2.576 | | 37.068 | | 24.562 | | 0.279 | |  |
| Kocide 3000 |  | 0.004 | | 0.152 | | 14.189 | | 0.096 | | 0.542 | 582.659 | | 24.551 | | 0.158 | | 0.005 | | 2.568 | | 31.419 | | 21.890 | | 0.458 | |  |
| Cu-EBDC |  | ND | | 0.161 | | 18.591 | | 0.110 | | 0.726 | 549.313 | | 26.649 | | 0.181 | | 0.008 | | 2.948 | | 37.159 | | 23.926 | | 0.265 | |  |
| Water |  | 0.012 | | 0.149 | | 14.230 | | 0.094 | | 0.564 | 487.194 | | 22.895 | | 0.155 | | 0.012 | | 2.431 | | 31.692 | | 23.098 | | 0.245 | |  |
| Significance |  | | NS | | NS | | Yes | | NS | NS | | NS | | NS | | NS | | NS | | NS | | NS | | NS | | NS | |
| Peel^z^ | | | | | | | | | | | | | | | | | | | | | | | | | | | |
| 20nm MgO | 1,000 | | 0.004 | | 0.144 | | 24.754 | | 0.083 | 1.039 | | 582.881 | | 25.357 | | 0.153 | | 0.005 | | 2.340 | | 37.557 | | 23.691 | | 0.250 | |
| 20nm MgO | 200 | | 0.003 | | 0.164 | | 21.535 | | 0.086 | 0.747 | | 586.713 | | 28.784 | | 0.158 | | 0.009 | | 2.430 | | 38.508 | | 23.684 | | 0.284 | |
| 0.3µm MgO | 1,000 | | 0.054* | | 0.197 | | 31.740* | | 0.120 | 0.948 | | 661.533 | | 36.165 | | 0.229 | | 0.012 | | 2.998 | | 50.027 | | 28.946 | | 0.369 | |
| 0.3µm MgO | 200 | | ND | | 0.171 | | 28.191 | | 0.093 | 0.722 | | 550.789 | | 29.672 | | 0.166 | | 0.012 | | 2.595 | | 37.675 | | 23.916 | | 0.275 | |
| 0.6µm MgO | 1,000 | | 0.006 | | 0.162 | | 25.043 | | 0.087 | 0.605 | | 565.484 | | 26.707 | | 0.152 | | 0.006 | | 2.448 | | 33.909 | | 22.637 | | 0.271 | |
| 0.6µm MgO | 200 | | 0.008 | | 0.151 | | 24.047 | | 0.080 | 0.824 | | 551.805 | | 26.898 | | 0.151 | | 0.006 | | 2.140 | | 32.540 | | 20.690 | | 0.750 | |
| Kocide 3000 |  | | 0.008 | | 0.145 | | 20.823 | | 0.081 | 0.512 | | 528.414 | | 24.825 | | 0.135 | | 0.005 | | 2.231 | | 29.526 | | 19.826 | | 0.364 | |
| Cu-EBDC |  | | 0.007 | | 0.176 | | 29.563* | | 0.105 | 0.901 | | 580.728 | | 32.292 | | 0.181 | | 0.011 | | 2.850 | | 39.375 | | 25.176 | | 0.295 | |
| Water |  | | 0.001 | | 0.138 | | 17.303 | | 0.081 | 0.553 | | 453.683 | | 23.113 | | 0.146 | | 0.008 | | 2.160 | | 31.662 | | 21.140 | | 0.228 | |
| Significance |  | | Yes | | NS | | Yes | | NS | NS | | NS | | NS | | NS | | NS | | NS | | NS | | NS | | NS | |
| Flesh | | | | | | | | | | | | | | | | | | | | | | | | | | | |
| 20nm MgO | 1,000 | | ND | | 0.129 | | 11.649 | | 0.090 | 0.797 | | 510.127 | | 19.835 | | 0.158 | | 0.006 | | 2.277 | | 32.574 | | 21.675 | | 0.226 | |
| 20nm MgO | 200 | | ND | | 0.126 | | 7.688 | | 0.082 | 0.565 | | 460.139 | | 19.336 | | 0.139 | | 0.006 | | 2.084 | | 28.484 | | 20.127 | | 0.237 | |
| 0.3µm MgO | 1,000 | | 0.025 | | 0.168 | | 12.155 | | 0.122 | 0.749 | | 548.389 | | 25.330 | | 0.217 | | 0.012 | | 2.855 | | 38.971 | | 26.100 | | 0.350 | |
| 0.3µm MgO | 200 | | ND | | 0.129 | | 9.293 | | 0.091 | 0.517 | | 424.362 | | 18.934 | | 0.153 | | 0.010 | | 2.188 | | 28.879 | | 19.645 | | 0.217 | |
| 0.6µm MgO | 1,000 | | 0.012 | | 0.137 | | 9.571 | | 0.090 | 0.488 | | 478.381 | | 20.045 | | 0.145 | | 0.006 | | 2.391 | | 29.060 | | 20.021 | | 0.239 | |
| 0.6µm MgO | 200 | | 0.002 | | 0.136 | | 8.449 | | 0.086 | 0.653 | | 517.514 | | 20.730 | | 0.154 | | 0.006 | | 2.132 | | 30.405 | | 20.976 | | 0.229 | |
| Kocide 3000 |  | | 0.014 | | 0.121 | | 6.465 | | 0.082 | 0.395 | | 427.854 | | 16.660 | | 0.123 | | 0.005 | | 2.091 | | 23.436 | | 17.274 | | 0.369 | |
| Cu-EBDC |  | | 0.001 | | 0.132 | | 9.119 | | 0.099 | 0.646 | | 446.044 | | 19.525 | | 0.161 | | 0.009 | | 2.490 | | 29.791 | | 20.543 | | 0.225 | |
| Water |  | | 0.001 | | 0.118 | | 9.541 | | 0.081 | 0.481 | | 385.744 | | 17.932 | | 0.132 | | 0.007 | | 1.966 | | 26.315 | | 18.655 | | 0.199 | |
| Significance |  | | NS | | NS | | NS | | NS | NS | | NS | | NS | | NS | | NS | | NS | | NS | | NS | | NS | |

^z^ In addition to whole-fruit samples, flesh, and peel samples were also collected separately. All of the samples were evaluated with Inductively Coupled Plasma Optical Emission Spectroscopy for elemental accumulation.

^x^ The concentration of 1 mg of copper in 1 kg of fresh weight tomato = 1 mg/kg FW Cu.

^y^An asterisk (*) indicates significant difference compared with water treatment (P = 0.05) based on Student-Newman-Keuls statistical analysis using the IBM SPSS program; NS = not significant.

^w^ND is not detected, concentration is below the limit of detection

^v^Cu-EBDC is composed of Kocide 3000 (2,100 µg/ml) and Penncozeb® 75DF (1,200 µg/ml).
